# The Clinical Role of Euthymia in Mental Health

**DOI:** 10.32872/cpe.14349

**Published:** 2024-06-28

**Authors:** Jenny Guidi

**Affiliations:** 1 Department of Psychology ‘Renzo Canestrari’, University of Bologna, Bologna, Italy; Philipps-University of Marburg, Marburg, Germany

The concept of euthymia was defined in the psychiatric literature essentially in negative terms, and extensively used to refer to the absence of mood disturbances meeting the threshold for a mental disorder based on diagnostic criteria or on cut-off points of dimensional assessment tools. However, in both unipolar and bipolar mood disorders, psychological distress may fluctuate considerably over time, even during the so-called ‘euthymic’ periods, and residual symptoms appear to be exceedingly common, indicating that the disorder is still present even though its intensity may vary (Dunner, 1999; Fava, 1999; Judd et al., 2002). This, in turn, could negatively impact on vulnerability to relapse in the long term (Fava, 1999).

## The Concept of Euthymia

In 2016, Fava and Bech introduced a novel definition of *euthymia*, according to specific clinimetric criteria, as a state characterized by the lack of mood disturbances that can be subsumed under diagnostic rubrics and the presence of features such as positive affect and balanced levels in psychological well-being dimensions (namely, autonomy, environmental mastery, positive relations with others, personal growth, purpose in life and self-acceptance), leading to flexibility, consistency, and resistance to stress (i.e., anxiety- or frustration-tolerance and resilience) (Fava & Bech, 2016). These psychological well-being dimensions, that were derived from Marie Jahoda’s model of positive mental health (Jahoda, 1958), have a bipolar nature, ranging from suboptimal to excessively elevated levels, and interact with each other through a compensatory, or eventually dysfunctional, dynamic balance.

This unifying concept of euthymia was subsequently refined by Fava and Guidi (2020) and further elaborated (Guidi & Fava, 2020, 2022), with particular regard to its relationships with other clinical constructs (e.g., individual characteristics, concurrent distress and other psychological attributes), and its associations with lifestyle behaviors and allostatic load. Allostatic load can be conceived as a result of the cumulative effects of daily life experiences encompassing both major challenges (i.e., life events) and ordinary events (i.e., subtle and/or repeated sources of chronic stress) (McEwen & Stellar, 1993). When environmental challenges exceed the individual ability to cope, then allostatic overload ensues, with a number of negative consequences on both physical and mental health (Fava et al., 2019; Guidi et al., 2021). According to McEwen’s (2020) viewpoint, ‘euthymia means using allostatis optimally and maintaining a healthy balance that promotes positive aspects of brain and body health through health-promoting behaviors’. Such behaviors include not only healthy eating habits, but also adequate and good-quality sleep, regular physical activity, refraining from smoking, alcohol and drug consumption, as well as positive social interactions and features of physical environment, which contribute to reduce allostatic load.

## Clinical Assessment of Euthymia

Exclusive reliance on diagnostic criteria (i.e., DSM-5-TR, ICD-11) often does not allow to capture the complexity of several manifestations that are encountered in clinical practice. A comprehensive clinical assessment requires appropriate collection and integration of a number of clinical variables, according to a clinimetric framework (Fava, 2022). Clinical interviewing should encompass evaluation of euthymia (Guidi & Fava, 2022), allostatic load/overload (Fava et al., 2019; Guidi & Fava, 2022) and lifestyle habits according to a longitudinal perspective. Instruments for assessing euthymia according to clinimetric principles have been developed. The Clinical Interview for Euthymia (CIE; Guidi & Fava, 2022) is a 22-item observer-rated clinimetric tool for gathering information on positive affect, both impaired and excessive levels in psychological well-being dimensions, flexibility, consistency and resistance to stress. Further, the Semi-Structured Interview for the Diagnostic Criteria for Psychosomatic Research (SSI-DCPR; Guidi & Fava, 2022) permits to assess, among other psychosomatic syndromes, allostatic overload and related constructs, such as demoralization.

As to self-rated clinimetric measures, the 10-item Euthymia Scale (ES; Fava & Bech, 2016; Carrozzino et al., 2019) represents an expanded version of the WHO-5 Well-Being Index (WHO-5; Topp et al., 2015) to evaluate positive affect and Jahoda’s well-being dimensions (i.e., flexibility, consistency and resistance to stress). Other clinimetric instruments that may be used jointly are the PsychoSocial Index (PSI; Piolanti et al., 2016) encompassing aspects related to allostatic load, and the Symptom Questionnaire (SQ; Benasi et al., 2020) for assessing both distress symptoms and well-being.

## Euthymia as a Treatment Target

Euthymia can be regarded as a therapeutic target, particularly when pre-planned sequential treatment strategies are implemented (e.g., psychotherapy after pharmacotherapy, or the sequential combination of two psychotherapeutic strategies) to decrease vulnerability to relapse in affective disorders, increase the level of recovery, and modulate mood (Guidi & Fava, 2021).

Well-Being Therapy (WBT; Fava, 2016; Guidi & Fava, 2020) is a manualized, short-term psychotherapeutic approach specifically aimed at modulating psychological well-being and pursuing a state of euthymia. WBT has introduced a clinical revolution in self-observation: patients are encouraged to identify episodes of well-being and their situational contexts. This systematic monitoring of well-being by using a structured diary represents a key, distinct therapeutic ingredient of WBT (Fava, 2016; Guidi & Fava, 2020), facilitates interaction between patients and therapists, and stimulates cognitive restructuring and homework (e.g., optimal experiences) based on the individual’s account and material.

Psychotherapeutic strategies geared to euthymia, such as WBT, should be applied within a clinimetric framework, based on clinical reasoning and case formulation according to macro- and micro-analysis, and staging (Fava, 2022). The treatment plan should be filtered by clinical judgment and integrate a number of clinical variables, such as severity and features of psychiatric disturbances, co-occurring symptoms and problems, medical comorbidities, patient’s history and preferences, and psychological well-being (Fava, 2022).

## Clinical Applications and New Developments

There are several potential clinical applications of treatments that target euthymia, including relapse prevention in depressive disorders, improving recovery in affective and other psychiatric disorders, modulating mood in bipolar-spectrum disorders, managing treatment resistance as well as discontinuation of psychotropic drugs, treating suicidal behavior and post-traumatic stress disorder (Guidi & Fava, 2020). Further, more recent findings support the clinical relevance of euthymia and lifestyle modification in improving medical outcomes, particularly in the setting of chronic medical diseases (Rafanelli et al., 2020). Indeed, a personalized approach targeting psychological well-being and euthymia may effectively improve patients’ health attitudes and behavior, and promote enduring lifestyle changes (Fava et al., 2023). Potential technical developments of WBT may derive from its application to couples, families, and groups, whereas computerized/digital methods of delivery could also be feasible, yet to be tested.

## Conclusions

The clinical role of euthymia supports innovative approaches to the assessment and treatment of mental disorders, and provides new, important insights in their psychotherapeutic management by modifying customary psychiatric approach, still unbalanced towards psychological dysfunction.

## Related Versions

This Editorial is based on Prof. Jenny Guidi’s oral presentation at the EACLIPT x UMH Conference 2024 in Amsterdam.
